# Highly specific PCR-RFLP assays for karyotyping the widespread 2Rb inversion in malaria vectors of the *Anopheles gambiae* complex

**DOI:** 10.1186/s13071-019-3877-x

**Published:** 2020-01-10

**Authors:** Raquel Montanez-Gonzalez, Verena Pichler, Maria Calzetta, Rachel R. Love, Alexandra Vallera, Lydia Schaecher, Beniamino Caputo, Marco Pombi, Vincenzo Petrarca, Alessandra della Torre, Nora J. Besansky

**Affiliations:** 1 Eck Institute for Global Health, & Department of Biological Sciences, University of Notre Dame, Notre Dame, IN 46556 USA; 2 Dipartimento di Sanità Pubblica e Malattie Infettive, Università “La Sapienza”, Istituto Pasteur-Fondazione Cenci-Bolognetti, 00185 Rome, Italy

**Keywords:** *Anopheles gambiae* complex, Chromosomal inversion, Inversion genotyping, Malaria vector, Molecular karyotyping, PCR-RFLP, Tag SNP

## Abstract

**Background:**

Chromosomal inversion polymorphisms play a role in adaptation to heterogeneous environments. Inversion polymorphisms are implicated in the very high ecological flexibility of the three main malaria vector species of the Afrotropical *Anopheles gambiae* complex, facilitating the exploitation of anthropogenic environmental modifications and promoting a strong association with humans. In addition to extending the species’ spatial and temporal distribution, inversions are associated with epidemiologically relevant mosquito behavior and physiology, underscoring their medical importance. We here present novel PCR-RFLP based assays strongly predictive of genotype for the cosmopolitan 2Rb inversion in *An. coluzzii* and *An. gambiae,* a development which overcomes the numerous constraints inherent to traditional cytological karyotyping.

**Methods:**

We designed PCR-RFLP genotyping assays based on tag SNPs previously computationally identified as strongly predictive (> 95%) of 2Rb genotype. We targeted those tags whose alternative allelic states destroyed or created the recognition site of a commercially available restriction enzyme, and designed assays with distinctive cleavage profiles for each inversion genotype. The assays were validated on 251 *An. coluzzii* and 451 *An. gambiae* cytologically karyotyped specimens from nine countries across Africa and one *An. coluzzii* laboratory colony.

**Results:**

For three tag SNPs, PCR-RFLP assays (denoted *Dra*III, *MspA*I, and *Tat*I) reliably produced robust amplicons and clearly distinguishable electrophoretic profiles for all three inversion genotypes. Results obtained with the *Dra*III assay are ≥ 95% concordant with cytogenetic assignments in both species, while *MspA*I and *Tat*I assays produce patterns highly concordant with cytogenetic assignments only in *An. coluzzii* or *An. gambiae*, respectively. Joint application of species-appropriate pairs of assays increased the concordance levels to > 99% in *An. coluzzii* and 98% in *An. gambiae*. Potential sources of discordance (e.g. imperfect association between tag and inversion, allelic dropout, additional polymorphisms in the restriction target site, incomplete or failed restriction digestion) are discussed.

**Conclusions:**

The availability of highly specific, cost effective and accessible molecular assays for genotyping 2Rb in *An. gambiae* and *An. coluzzii* allows karyotyping of both sexes and all developmental stages. These novel tools will accelerate deeper investigations into the role of this ecologically and epidemiologically important chromosomal inversion in vector biology.
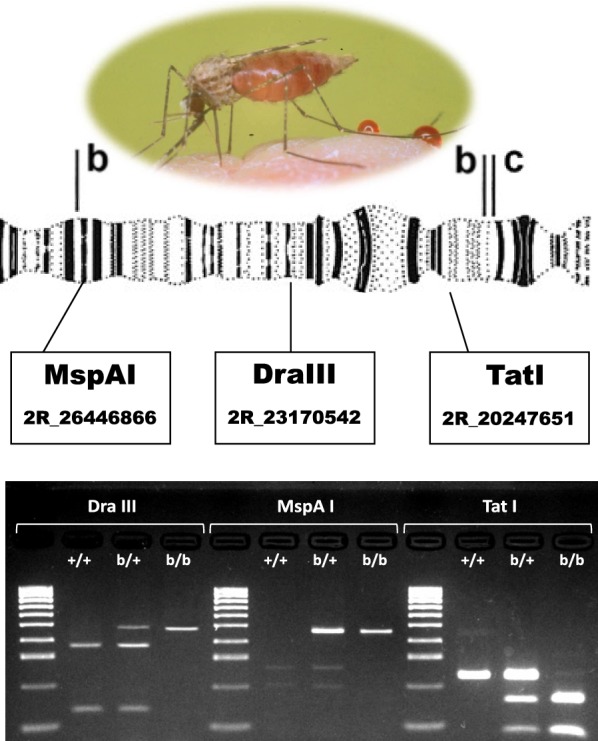

## Background

The three main malaria vector species belonging to the Afrotropical *Anopheles gambiae* complex, *An. coluzzii*, *An. gambiae* and *An. arabiensis*, are characterized by extensive paracentric inversion polymorphisms mostly involving the right arm of chromosome 2 [1, 2]. By suppressing recombination between alternative arrangements in heterokaryotypes and maintaining blocks of locally adapted genes within the breakpoints [3, 4], these paracentric inversions have enabled an extraordinary ecological flexibility, allowing colonization of different habitats across sub-Saharan Africa and facilitating ecological differentiation [5–8]. Inversion polymorphisms also are implicated in the efficient exploitation of anthropogenic environmental modifications and ecological disturbances such as irrigation and deforestation [1, 2, 9–13], helping to promote a strong association between these mosquitoes and humans. This has not only extended their spatial and temporal distribution but also helped transform these species into the most efficient malaria vectors worldwide.

The adaptive value of inversion polymorphisms is particularly evident in the case of the 2La arrangement in *An. coluzzii* and *An. gambiae,* whose temporal and spatial distribution is strongly correlated with degree of aridity [2, 14–16]. This strong correlation was first shown 40 years ago based on the demanding cytological karyotyping of thousands of polytene chromosome slides obtained from ovarian nurse cells of females at the half-gravid stage after blood meal - the only adult stage with sufficient chromosome polytenization to allow examination of the banding pattern [17]. Only subsequent to the relatively recent molecular characterization of the 2La breakpoint regions [18] did it become possible to develop a reliable PCR-based diagnostic assay [19] that made karyotyping accessible to non-cytogeneticist and allowed the scoring of large numbers of specimens irrespective of gender, life stage, physiological status, or method of specimen storage. Since then, application of this assay has facilitated the study of ecologically relevant phenotypes associated with the 2La inversion in both laboratory and field settings, such as enhanced desiccation resistance and response to thermal stress [20–26]. Initial cytogenetic observations made decades ago also associated inversion 2La with phenotypes of epidemiological importance, such as susceptibility to *Plasmodium* infection [27], indoor resting behaviour and response to vector control interventions [2]. Development of molecular diagnostics for inversions like 2La overcomes an important obstacle to follow-up association studies [28] that have been unfeasible before now. Future applications of this assay and others under development for additional inversions will foster a deeper understanding of already recognized or suspected phenotypic associations, and boost the discovery and dissection of unsuspected physiological and behavioural traits of epidemiological and ecological relevance determined by inversions.

Non-random spatial and temporal distribution with respect to degree of aridity also characterizes inversion frequencies on the right arm of chromosome 2, where up to five common inversion polymorphisms segregate in *An. coluzzii* and *An. gambiae*. Here, we focus on 2Rb because, aside from 2La, it is the only other inversion in these species with a cosmopolitan distribution across sub-Saharan Africa [1]. Despite molecular characterization of its breakpoints, complex repetitive flanking sequence precluded the development of a robust PCR-based karyotyping assay for this inversion *via* the same breakpoint-spanning strategy used for the 2La inversion [29]. The breakpoint-proximal 2Rb PCR diagnostic that was developed is not applicable for natural populations due to poor accuracy [29]. Without modern molecular tools that are widely accessible, current understanding of the phenotypic and epidemiological significance of the 2Rb inversion is largely limited to a few historical cytogenetic studies, mostly focused on the association of this polymorphism with dry environments or seasons [2, 13, 27, 30]. The same 2Rb inversion is polymorphic in *An. arabiensis*, where it has been associated with host-choice [27, 31], underscoring its broader epidemiological relevance in the *An. gambiae* complex and the importance of studying it more closely.

Recently, multiple tag single nucleotide polymorphisms (SNPs) significantly associated with inversions across geography were computationally identified [32] in a database of genomic variation (Ag1000G) based on deep genome re-sequencing of thousands of specimens from natural *An. coluzzii* and *An. gambiae* populations spanning Africa [33]. These tag SNPs are suitable for *in silico* karyotyping of individual fully sequenced *An. gambiae* and *An. coluzzii* mosquitoes (not *An. arabiensis*, as it was underrepresented in Ag1000G at the time of tag ascertainment). They are also under development as tools for high throughput molecular karyotyping of unsequenced mosquitoes, using targeted approaches such as amplicon sequencing [32]. However, the need remains for inexpensive and widely accessible approaches for genotyping of individual inversions. Amplicon sequencing is ideally suited to large-scale studies, which may not serve more focused needs or smaller budgets. Equally important, those planning to embark on major GWAS studies using amplicon sequencing for inversion genotyping will need to make sure ahead of their sequencing investment that the inversions of interest are sufficiently polymorphic in their populations to give them adequate power to find significant associations if they exist, a goal well-suited to inexpensive PCR assays.

Here we present novel PCR-RFLP based assays that exploit three of the SNPs previously identified [32] as strongly predictive of 2Rb inversion status in *An. coluzzii* and *An. gambiae*. We validated these assays on hundreds of cytologically karyotyped *An. coluzzii* and *An. gambiae* samples collected across Africa. These assays fill an important gap in available resources required to further our understanding of behavioural, physiological, and epidemiological traits conferred by this widespread inversion, potentially revealing heterogeneities relevant to the success of vector control interventions.

## Methods

### Cytological karyotyping

*Anopheles coluzzii* and *An. gambiae* field-collected specimens were molecularly identified and cytologically karyotyped either specifically for this study or in the framework of previously published studies (Additional file 1: Table S1). In addition, *An. coluzzii* specimens from the Banfora M colony were karyotyped. This colony was established in 2014 from collections made in the Banfora District of Burkina Faso by the Liverpool School of Tropical Medicine and Hygiene with support from the Centre National de Recherche et de Formation sur le Paludisme. Polytene chromosome preparations followed della Torre [17], extending the hydration of ovarian follicles up to 4 h where necessary, to compensate for the several years of preservation in Carnoy’s solution for historical samples. Paracentric inversion karyotypes were scored according to established nomenclature [2, 13]. All chromosomal slides specifically prepared as part of this study were karyotyped by two independent experts and polytene complements were documented with photomicrographs. Micrographs were retained to allow reassessment of the cytogenetic karyotype in the event of incongruent cytogenetic and molecular results.

### Design of PCR-RFLP genotyping assays for 2Rb

Tag single nucleotide polymorphisms (SNPs) predictive of 2Rb genotype were computationally identified previously [32]. Briefly, fully sequenced specimens of *An. coluzzii* and *An. gambiae* from the Ag1000G database of natural variation [33] were assigned a presumptive 2Rb inversion genotype using local principal components analysis (PCA) of biallelic SNPs in a window of the genome corresponding to 2Rb. Tag SNPs in that window were those whose genotypes were highly concordant with PCA-based inversion genotypes, such that for most specimens (> 80%), the number of alternate alleles at that site (0, 1 or 2) matched the number of chromosomes inverted for 2Rb carried by the corresponding specimen (0, 1 or 2). For the purposes of designing robust PCR-RFLP genotyping assays from a small subset of the resulting 349 tag SNPs identified by Love et al. [32], we began with the ten tags that showed the highest degree of concordance (> 95%) between SNP- and inversion genotype. Among these ten tag SNPs, we screened for those in which alternative allelic states destroyed or created a restriction enzyme recognition site cleavable by a commercially available enzyme (*n* = 5), using RestrictionMapper v3 software [34]. Using the *An. gambiae* and *An. coluzzii* reference genomes (AgamP4 and AcolM1, respectively) accessed through VectorBase [35] and Primer3Plus v.2.4.2 software [36], we designed primer pairs expected to anneal in both species, that flanked each tag SNP and produced amplicons 200–500 bp in length. We avoided primer binding sites containing either high frequency variants (> 5%, as judged from Ag1000G variation data) or repetitive sequence (as judged from softmasking of AgamP4). We prioritized those assays with robust amplification and enzyme cleavage, and whose electrophoretic profiles provided optimal contrast between inversion genotypes.

### PCR-RFLP genotyping

Genomic DNA extraction was conducted from individual cytogenetically karyotyped specimens following a variety of standard protocols, including DNAzol Reagent (Thermo Fisher Scientific, Waltham, MA, USA), DNeasy Blood & Tissue Kit (Qiagen, Hilden, Germany), CTAB, and other approaches. Concentration and quality of a subset of genomic DNA samples was assessed using Quant-iT PicoGreen dsDNA Reagent (Thermo Fisher Scientific) or the Nanodrop 2000 spectrophotometer (Thermo Fisher Scientific). The mean concentration was 26 ng/µl based on PicoGreen quantification.

PCR was carried out in 25 µl reactions containing 20 mM Tris-HCl (pH 8.3), 50 mM KCl, 200 µM of each dNTP, 2 mM MgCl_2_, 5–10 pmol of each primer, 1 U of Taq polymerase and 1 µl of template genomic DNA. PCR conditions included an initial incubation at 94 °C for 2 min, 35 cycles of 94 °C for 30 s, 58 °C for 30 s and 72 °C for 45 s, followed by 72 °C for 2 min and a 4 °C hold.

Restriction digests were performed in 20 µl reactions with 0.5 µl of the appropriate restriction enzyme, following manufacturer recommendations (*Dra*III and *MspA*I in 1× CutSmart Buffer at 37 °C for 1 h, (New England Biolabs, Ipswich, MA, USA); *Tat*I in 1× Tango Buffer at 65 °C for 1 h (ThermoFisher Scientific). The amount of PCR product added to each reaction varied from 5 µl for *Dra*III and *MspA*1 digests, to 8–10 µl for *Tat*I digests. Optionally, *Dra*III and *MspA*I digests were inactivated at 65 °C for 20 min. Results were analyzed by electrophoresis through agarose gels stained with SYBR Safe, using TBE buffer (2% agarose and 0.5× TBE at the University of Notre Dame; 3% agarose and 1× TBE at the University of Rome). Optionally, SDS loading dye was prepared (10 µl of 10%SDS per 1 ml of 6× loading dye) and added to samples prior to electrophoresis to eliminate protein-DNA interactions and prevent gel shifts, as recommended by Thermo Fisher Scientific.

### Amplicon sequencing

Enzymatic cleanup of the amplified PCR product was achieved in reactions containing 2 U of Exonuclease 1 (USB Corporation, Cleveland, OH), 1 U of Shrimp Alkaline Phosphate (USB Corporation), 1.8 µl of ddH2O, and 8 µl of the PCR product. After incubation at 37 °C for 15 min, the enzymes were inactivated at 80 °C for 15 min. Sanger sequencing was performed directly on the resulting samples, using one PCR primer and the ABI 3730X1 DNA Analyzer Platform (PE Applied Biosystems, Warrington, England).

## Results and discussion

**Fig. 1:**
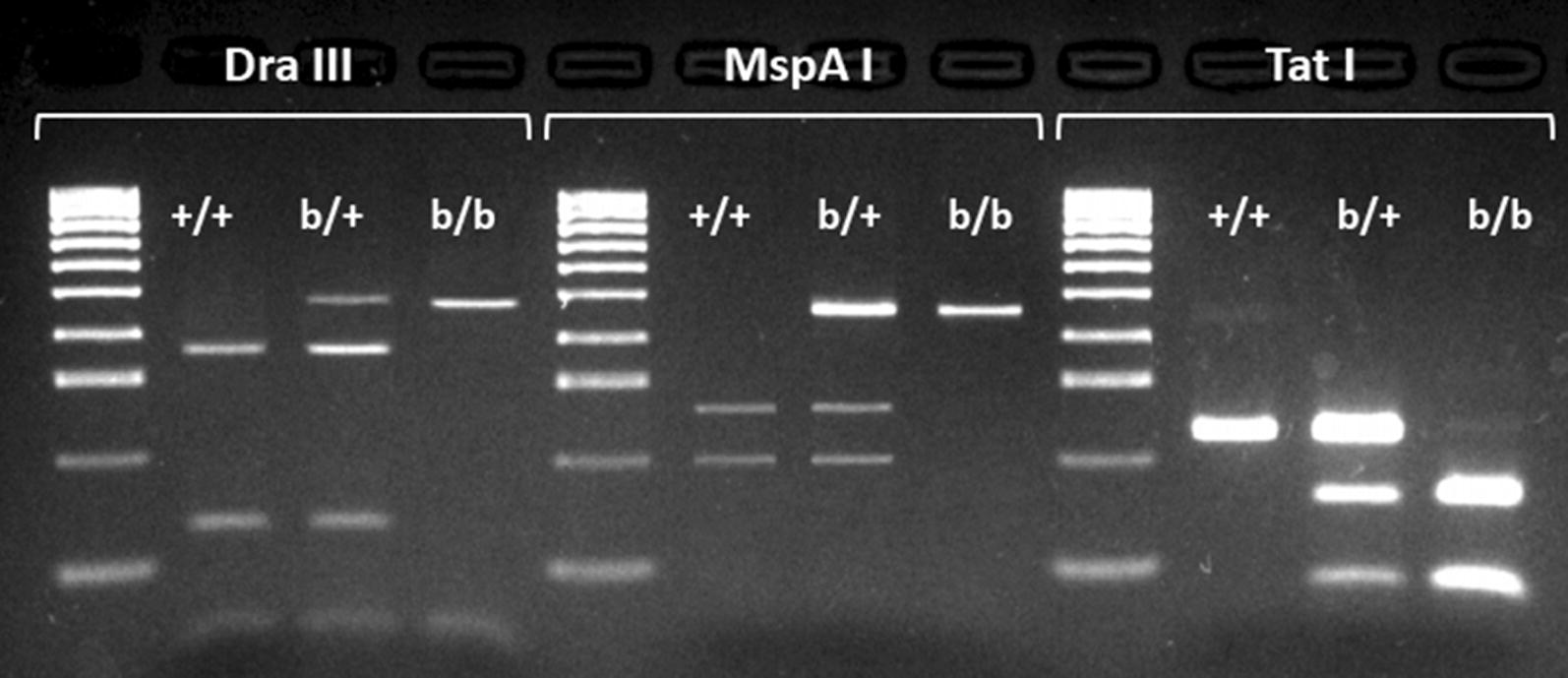
Representative electrophoretic profiles of the *Dra*III, *MspA*I and *Tat*I assays for inversion genotyping of 2Rb. Standard (un-inverted) homozygotes for 2Rb, +/+; heterozygotes, b/+; inverted homozyogtes, b/b. Molecular weight marker (Lanes 1, 5, 9), HyperLadder 100 bp (Bioline, Memphis, TN, USA): 100–1000 bp in increments of 100 bp

**Fig. 2:**
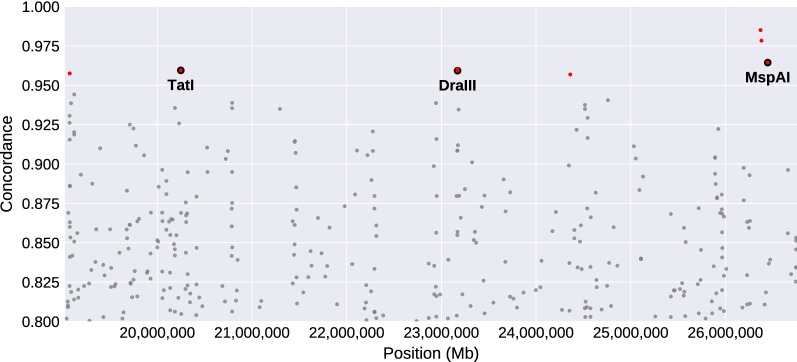
Position of tag SNPs within 2Rb. Scatterplot of genomic location and SNP genotype-inversion genotype concordance for tag SNPs identified for 2Rb. SNPs with concordance > 95% are in red. Those targeted by PCR-RFLP assays are circled and labelled by assay name

Of the 349 tag SNPs computationally identified as predictive of 2Rb genotype by Love et al. [32], we focused on those whose concordance with PCA-based inversion genotype in Ag1000G was > 95% and whose alternative alleles created or destroyed the recognition sequence of a commercially available restriction enzyme. For three of five such tags, it was possible to design PCR-RFLP assays that reliably produced robust amplicons and distinctive electrophoretic profiles for all three inversion genotypes (Table 1, Fig. 1). For simplicity and brevity, we refer to these three assays by the names of the restriction enzymes each assay employs: *Dra*III, *MspA*I and *Tat*I. The chromosomal location of the three tag SNPs targeted by each assay, shown in relation to the 2Rb inversion breakpoints and other 346 tags, is shown in Fig. 2. Overall, the set of 349 tags is not noticeably skewed toward inversion breakpoints, and one of the assay tags (*Dra*III) is centrally located inside the inversion. Each of the three assays was tested on cytologically karyotyped specimens sampled independently of Ag1000G, from nine countries across Africa (251 *An. coluzzii* and 451 *An. gambiae*), and one chromosomally polymorphic *An. coluzzii* laboratory colony recently established from Burkina Faso (Table 2, Additional file 1: Table S1).

**Table 1:** PCR-RFLP genotyping assays for inversion 2Rb in *An. gambiae* and *An. coluzzii*

Tag position	Concord	Ref/Alt	Restriction enzyme	Chromosome cut	Primer pairs (5’-3’)	Amplicon size (bp)	Cleavage products (bp)
23170542	96.7%	C/T	*Dra*III	2R+^b^	F: GCCGTTCTCCAGGCTCAG	481	348, 133
					R: AAACTCCATTGTACTGGCTGAA		
26446866	96.7%	G/A	*MspA*1	2R+^b^	F: TTCACAACGAAATGGCAAGA	463	264, 199
					R: TAGGGCAGTGTTGAGGGAAC		
20247651	96.3%	G/A	*Tat*I	2Rb	F: AATGGCCAGTCTCGAAAGAA	227	153, 74
					R: GAAGGGAACGTATGATAATGCAG		

*Abbreviations*: Tag position, chromosome coordinate; Concord, minimum percent concordance with inversion genotype based on Love et al. [32]; Ref/Alt, reference and alternate allele at tag SNP

**Table 2:** Degree of concordance between cytological karyotype and individual PCR-RFLP genotyping assay

Country	*Dra*III concordance (%)		*MspA*I concordance (%)		*Tat*I concordance (%)	
	*An. coluzzii*	*An. gambiae*	*An. coluzzii*	*An. gambiae*	*An. coluzzii*	*An. gambiae*
Benin	19/19 (100)	na	19/19 (100)	na	15/19 (78.9)	na
Burkina Faso	na	19/20 (95.0)	na	14/20 (70)	na	17/20 (85)
Burkina Faso (lab)	25/25 (100)	na	26/27 (96.3)	na	16/24 (66.6)	na
Cameroon	10/10 (100)	263/276 (95.2)	10/10 (100)	208/274 (75.9)	10/10 (100)	253/275 (92)
Gambia + Senegal^a^	6/6 (100)	56/58 (96.6)	5/6 (83.3)	52/56 (92.9)	4/6 (66.7)	47/47 (100)
Guinea Bissau	3/3 (100)	18/18 (100)	3/3 (100)	16/16 (100)	3/3 (100)	16/16 (100)
Mali	201/211 (95.3)	55/57 (96.5)	128/131 (97.7)	22/26 (84.6)	104/132 (78.8)	21/28 (75)
Tanzania + DRC^a^	na	11/13 (84.6)	na	13/13 (100)	na	13/13 (100)
Total	264/274 (96.4)	422/443 (95.3)	191/196 (97.4)	325/405 (80.2)	152/194 (78.3)	367/399 (92.0)

^a^Specimens were combined into one sample due to geographical proximity and small sample size

*Abbreviations*: DRC, Democratic Republic of Congo; na, not available

### Sources of discordance and their mitigation

Before detailing the results of each assay, we first consider the factors that could produce disagreement between cytogenetic and PCR-RFLP evidence, and the approaches we took to limit this where feasible. Although we predicted strong agreement between cytological and PCR-RFLP genotype assignments on the basis of > 95% concordance between the component tag SNPs and 2Rb inversion status in Ag1000G (Table 1), the association between tag and inversion is inherently imperfect. Given this unavoidable limitation, multiple PCR-RFLP assays can be combined on the same specimen to increase confidence in the genotypic assignment (see below). In addition, evidence from Table 3 of Love et al. [32] suggests that the rate of human error in 2Rb cytogenetic karyotyping and/or metadata recording is ~4%. We were able to address this issue for the cytogenetic karyotyping performed specifically for this study, by preserving slides that were used to make assignments as well as by preparing an extensive photomicrographic record, allowing us to re-examine (confirm) the cytological assignments in the event of disagreements. This was possible for 227 specimens, but not for the remaining specimens that were processed during preceding studies that did not take the same precautionary measures. Finally, the PCR-RFLP process may also produce artifactual results for technical reasons or due to genetic polymorphisms. The possibility of incomplete or failed restriction digestion is a technical issue that we mitigated by repeating PCR-RFLP assays in the presence of controls, whenever genotypic mismatches were encountered. Substitutions elsewhere in the restriction enzyme recognition site, even if the allelic state of the tag matches the enzyme recognition sequence, can prevent enzymatic cleavage. To determine whether conflicting cytogenetic and PCR-RFLP assignments could be attributed to non-focal (i.e. non-tag) nucleotide polymorphisms in the enzyme recognition sites, we sequenced a subset of PCR amplicons (*n* = 80). When designing PCR primers flanking the tag SNP, we avoided known polymorphic sites with frequencies $\ge$ 5% in Ag1000G, but in highly polymorphic species like *An. gambiae* and *An. coluzzii* [33], the occurrence of polymorphisms in the primer binding sites that could prevent or hinder primer annealing and extension in a fraction of specimens is plausible, and could lead to underrepresentation or elimination of the affected allele (‘allelic dropout’). Allelic dropout, commonly observed in microsatellite data from a broad variety of organisms including *An. gambiae* [37, 38], is manifested by the underrepresentation of heterozygotes in a population sample. Genetic evidence of a heterozygote deficit typically comes from tests of Hardy-Weinberg equilibrium (HWE), but in the present study, application of this test is complicated by small sample sizes, sourced from many distinct localities even within the same country, challenging the assumption of HWE. Direct evidence (and mitigation) of allelic dropout by designing and applying alternative primers, is a viable but labor-intensive option not adopted here.

### *Dra*III

The overall rate of concordance between the *Dra*III assay and cytological karyotypes was comparably high in both species, 96.4% for *An. coluzzii* and 95.5% for *An. gambiae* (Table 2, Additional file 1: Table S1). This performance is not substantially different from the degree of concordance between the tag SNP and inversion status in the Ag1000G database (96.7%; Table 1). The small total number of discordant cytogenetic and *Dra*III assignments can be explained at least in part by the fact that the association between tag and inversion is imperfect.

We considered other sources of discordance between *Dra*III and cytogenetics among the 10 *An. coluzzii* and 20 *An. gambiae* specimens with conflicting assignments (Additional file 1: Table S1). Allelic dropout is the most plausible explanation for the five *An. coluzzii* and 12 *An. gambiae* in which a cytogenetically heterozygous karyotype (‘1’) disagreed with a *Dra*III homozygous profile (‘0’ or ‘2’). Moreover, in the 12 instances in which cytogenetic heterozygotes in either species were classified by *Dra*III as homozygous inverted (‘2’), another (not mutually exclusive) explanation could be failure of enzymatic digestion of true 2R+^b^ amplicons, either for technical reasons or due to the presence of additional SNPs in the recognition sequence apart from the tag itself. Sequencing of five *An. gambiae* amplicons from specimens typed as ‘1’ cytogenetically and as ‘2’ by their *Dra*III profiles revealed no evidence of sequence heterozygosity at the tag SNP position, as would have been expected for a true heterozygote. While we cannot rule out that we may have failed to detect true heterozygotes due to strong allelic imbalance in the sequencing reaction, all amplicon sequences appeared to be homozygous at the tag SNP for the uncleavable ‘2’ allele. This suggests that the discrepancies are not due to technical problems with restriction digestion, but more likely are due to allelic dropout and/or incomplete association of the tag with the inversion. Interestingly, in one of these five specimens we did detect a different polymorphism in the *Dra*III recognition site apart from the tag position, but because the genotype at the tag already rendered it uncleavable by *Dra*III, this substitution did not impact the expected *Dra*III profile.

We also sequenced four amplicons from *An. coluzzii* and *An. gambiae* derived from specimens whose cytogenetic assignment was homozygous ‘0’, but whose *Dra*III profile was heterozygous. In one case, sequencing confirmed the cytogenetic assignment, revealing another SNP in the *Dra*III recognition sequence of one allele that explained the *Dra*III restriction profile of ‘1’. For two other specimens, sequencing validated the *Dra*III profile, a result consistent with incomplete association of the tag with the inversion or with partial digestion. The fourth specimen with an unconfirmed cytological assignment of ‘2’ had a *Dra*III profile of ‘1’, but sequencing revealed that the tag SNP genotype was ‘0’, with no indication of additional SNPs in the recognition sequence. The underlying conflict between cytology (‘2’) and sequence (‘0’) is unresolved, but the *Dra*III profile of ‘1’ is consistent with partial digestion.

### *MspA*I

In our previous work, the 349 tag SNPs developed for 2Rb proved highly concordant with inversion status in both species and worked well for *in silico* karyotyping regardless of taxon [32]. It therefore surprised us initially that in the present study, the performance of the *MspA*I assay depended strongly on taxonomic status (Table 2, Additional file 1: Table S1). Whereas the agreement between cytological and *MspA*I assignments was 97.4% for *An. coluzzii*, with only five specimens showing mismatches, much lower agreement (80.2%) was measured for *An. gambiae*. Close scrutiny suggests that the *An. gambiae* discrepancies were most likely caused by allelic dropout rather than a failure of the tag SNP itself to predict inversion status. In fact, 66 of 80 *An. gambiae* specimens with discordant genotypic assignments (among 405 scored) had a cytogenetic karyotype of ‘1’ and a *MspA*I profile of ‘0’ or ‘2’. Moreover, 56 of those 66 had *MspA*I profiles of ‘2’, further suggesting that the standard (uninverted) chromosome was the more likely to be affected by allelic dropout. Sequence analysis of the amplicons from 34 *An. gambiae* specimens with discrepant *MspA*I profiles of ‘0’ (*n* =8) and ‘2’ (*n* = 26) revealed tag genotypes consistent with the *MspA*I assay.

We also sequenced representative amplicons of five *An. gambiae* specimens manifesting other discrepancies, in which a homozygous cytogenetic karyotype (‘0’ or ‘2’) disagreed with a heterozygous *MspA*I profile (two cytologically confirmed examples were sequenced), or an *MspA*I profile of the opposite homozygote (three examples were sequenced; cytological confirmation was lacking). Sequencing revealed no polymorphisms in the *MspA*I restriction site apart from the tag itself, and the tag status was fully concordant with the *MspA*I digestion profile.

For the five *An. coluzzii* (of 196 scored) with mismatches between cytogenetic and molecular profiles, the cytogenetic assignment was double-checked and confirmed in all cases. Three of these had a cytogenetic karyotype of ‘1’ accompanied by a homozygous *MspA*I profile of either ‘0’ or ‘2’. In all three, sequencing confirmed the homozyogous *MspA*I profile, with no additional SNPs in the recognition sites. The other two mismatches involved a cytologically homozygous karyotype (‘0’ or ‘2’) with a heterozygous *MspA*I profile that was confirmed by sequencing.

If it is assumed that the cytogenetic karyotype was the correct one in each of the above instances of conflict, allelic dropout is one possible explanation when a cytogenetic heterozygote assignment disagrees with a molecular homozygote assignment, but this possibility is smaller if the tag genotype is ‘1’. Whatever the conflict, incomplete association of the tag with the inversion is another, non-exclusive, explanation.

### *Tat*I

Overall concordance between cytogenetic karyotype and the *Tat*I assay was lower than for the other two assays, but as was the case for *MspA*I, there was also a pronounced difference between species. Agreement between cytogenetic and *Tat*I assignments was 92% for *An. gambiae*, but only 78.4% for *An. coluzzii*. Unlike the MspIA assay, discordances consistent with allelic dropout (i.e. cytogenetic assignment of ‘1’ and *Tat*I assignment of ‘0’ or ‘2’) were not disproportionate to other types of discordance in either species. Instead, *An. coluzzii* simply had a higher rate of conflicts of all types (Table 2, Additional file 1: Table S1).

Sequencing of amplicons from 15 specimens (12 *An. gambiae* and 3 *An. coluzzii*) with heterozygous cytogenetic assignments and homozygous *Tat*I profiles invariably confirmed that the tag SNP genotype matched the *Tat*I profile, and no other SNPs were identified in the restriction site, consistent with allelic dropout and/or incomplete association with the tag and the inversion.

Sequencing of amplicons from eight *An. gambiae* with homozygous cytogenetic assignments discordant with heterozygous *Tat*I profiles, revealed four cases in which the tag genotype agreed with the *Tat*I digestion profile. Of these, one could be explained by another SNP in the restriction site, and the remaining three implicated an incomplete *Tat*I digest leading to an inaccurate *Tat*I profile. In three *An. coluzzii* specimens with homozygous cytogenetic assignment and heterozygous *Tat*I profile, sequencing confirmed the *Tat*I profile, suggesting that if the cytological assignment is assumed correct, these represent the incomplete association of the tag with the inversion.

Finally, sequencing of the amplicon from one *An. gambiae* specimen with opposite homozygote assignments (cytogenetic ‘2’ *vs Tat*I profile ‘0’), confirmed the *Tat*I assignment, suggesting incomplete association between tag and inversion.

### Combinatorial approaches

The *Dra*III assay is ≥ 95% concordant with cytogenetic assignments in both species, a level that should be adequate for most applications. However, if additional confidence is desired, two assays could be applied jointly on the same specimen. This might be advisable for molecular karyotyping of mosquito populations from regions underrepresented in the Ag1000G database (at the time we accessed it for our work), or underrepresented in the present study, in which *An. gambiae* samples from Cameroon and *An. coluzzii* samples from Mali predominate.

Our data suggest that the combination of *Dra*III and *MspA*I for *An. coluzzii* (individually concordant with cytogenetics at 96.4% and 97.4%, respectively) and of *Dra*III and *Tat*I for *An. gambiae* (individually concordant at 95.5% and 92%) would be most effective. Joint application of these pairs increased the concordance between cytogenetic and molecular assignments to > 99% (185/186) in *An. coluzzii* and 98% (354/361) in *An. gambiae*. In practice, specimens with conflicting molecular assignments (6 of 192 for *An. coluzzii* and 29 of 390 for *An. gambiae*) would be considered ambiguous and should be excluded.

## Conclusions

Here we have developed three cost effective and accessible molecular assays that can be used individually or in combination for genotyping 2Rb in *An. gambiae* and *An. coluzzii* with high specificity. Their performance metrics are based on the conservative assumption that the cytogenetic karyotype is the correct one in the case of conflict between cytogenetic and molecular assignments. Indeed, our results suggest that a variety of phenomena (e.g. imperfect association between tag and inversion, allelic dropout, polymorphisms in the enzyme recognition and/or primer binding sites) contribute to incorrect molecular assignments. However, cytogenetic karyotyping is not infallible, and our experimental design allowed for the validation of only a fraction of the cytogenetic assignments used in this study. From the 1970s to the 1990s, a series of double-blind checks by cytogeneticists at the University of Rome La Sapienza (including the present authors) produced error estimates ranging from 0% to 5%, depending on slide quality. Errors were mainly due to mismatch between the actual reading and karyotype encoding, either on the preparation slide or on the recording sheets, rather than actual banding pattern misinterpretations (V. Petrarca, personal communication). Other groups with less extensive cytogenetic skill and experience might encounter higher error rates. Accordingly, the true accuracy of the PCR-RFLP assays may exceed what we report here. The *MspA*I assay performed relatively poorly in *An. gambiae* largely due to allelic dropout. Compared to the other two assays, *MspA*I targets a SNP very near one of the 2Rb breakpoints (Fig. 2), where the recombination rate is expected to be relatively low. Low recombination should enhance population structure, both between opposite orientations of 2Rb and between the two taxa. Future directions include designing PCR-RFLP assays to genotype 2Rb in *An. arabiensis*, once this species is adequately represented in Ag1000G. In addition, 2Rc is an inversion locally common in West Africa that, like 2Rb, is implicated in environmental adaptation and ecotypic differentiation. Based on the tag SNPs previously identified in *An. gambiae* and *An. coluzzii* [32], efforts are underway to develop PCR-RFLP assays for 2Rc genotyping. Together, these assays will accelerate deeper investigations into the role of these ecologically and epidemiologically important chromosomal inversions in vector biology.

## Supplementary information

**Additional file 1: Table S1.** Detailed genotypic concordance between cytological karyotype and individual PCR-RFLP genotyping assay.

## Data Availability

Data supporting the conclusions of this article are included in the article and its supplementary files. In addition, amplicon sequences determined from this study are available in GenBank under accession numbers MN599476-MN599555.
